# L-655,708 Does not Prevent Isoflurane-induced Memory Deficits in Old Mice

**DOI:** 10.1515/tnsci-2019-0032

**Published:** 2019-08-07

**Authors:** Teng Gao, Yue Liu, Zifang Zhao, Yuan Luo, Lifang Wang, Yongan Wang, Yiqing Yin

**Affiliations:** 1 Department of Anesthesiology, Beijing Shijitan Hospital, Capital Medical University, Beijing, 100038, China; 2 Department of Anesthesiology, China-Japan Friendship Hospital, Beijing, 100029, China; 3 State Key Laboratory of Toxicology and Medical Countermeasures, Academy of Military Medical Sciences, Beijing, 100850, China

**Keywords:** POCD, Old age, GABA, Inverse agonist, L-655,708

## Abstract

**Background:**

General anesthesia and increasing age are two main risk factors for postoperative cognitive dysfunction (POCD). Effective agents for the prevention or treatment of POCD are urgently needed. L-655,708, an inverse agonist of α5 subunit-containing γ-aminobutyric acid subtype A (α5GABA_A_) receptors, can prevent anesthesia-induced memory deficits in young animals. However, there is a lack of evidence of its efficacy in old animals.

**Methodology:**

Young (3- to 5-month-old) and old (18- to 20-month-old) mice were given an inhalation of 1.33% isoflurane for 1 hour and their associative memory was evaluated 24 hours after anesthesia using fear-conditioning tests (FCTs). To evaluate the effect of L-655,708, mice received intraperitoneal injections of L-655,708 (0.7 mg/kg) or vehicle 30 minutes before anesthesia.

**Results:**

Old mice exhibited impaired memory and lower hippocampal α5GABA_A_ levels than young mice under physiological conditions. Pre-injections of L-655,708 significantly alleviated isoflurane-induced memory decline in young mice, but not in old mice.

**Conclusions:**

L-655,708 is not as effective for the prevention of POCD in old mice as it is in young mice. The use of inverse agonists of α5GABA_A_ in preventing POCD in old patients should be carefully considered.

## Introduction

Life expectancy rises across the globe, and more and more elderly patients are undergoing surgery and receiving anesthesia. The incidence of postoperative cognitive dysfunction (POCD), which refers to a decline in learning and memory after surgery [1], is much higher in aged patients than in young patients [2, 3]. POCD not only decreases the life quality of patients, but also imposes a serious burden on healthcare [4], and there is to date no effective therapy for POCD. Thus, effective agents for the prevention or treatment of POCD are urgently needed.

The γ-aminobutyric acid subtype A (GABA_A_) receptor is the main target for most anesthetics [5]. GABA_A_ receptors are chloride-selective ion channels composed of multiple subunits (α1-6, β1-3, γ1-3, δ, ε, θ, π, ρ1-3) [6], and most GABA_A_ receptors consist of two α subunits, two β subunits and one γ subunit [6]. In particular, the α5 subunit-containing GABA_A_ (α5GABA_A_) receptor, which preferentially localizes in the hippocampus [7], plays an important role in memory and learning. The genetically deletion or reduction of α5GABA_A_ can improve cognitive function under physiological conditions [8, 9, 10]. Furthermore, the activation of α5GABA_A_ contributes to the amnestic effect of anesthetics and anesthesia-induced memory decline [11, 12, 13]. Therefore, inhibiting the α5GABA_A_ receptors has become a potential strategy to prevent or to treat POCD.

L-655,708 is an inverse agonist of α5GABA_A_, acts as a negative allosteric modulator (NAM) of α5GABA_A_ receptors [14]. Studies have suggested that L-655,708 not only enhances cognition under normal conditions [15], but also prevents anesthesia-induced memory deficits in rodents [13, 16, 17]. Most of these experiments were done in young rodents, and the evidence about the effect of L-655,708 in old animals remains lacking. Since the elderly patients are more susceptible to POCD than young patients, in current study, we sought to evaluate the efficacy of L-655,708 in preventing POCD in old animals.

## Materials and Methods

### Animals

All animal procedures were approved by the Animal Experimental Ethics Committee of the China-Japan Friendship Hospital (Beijing, China). 3- to 5-month-old (young) and 18-to 20-month-old (old) male C57/BL6 mice, which correspond to 20- to 30-year-old and 60- to 70-year-old human respectively [18], were purchased from Vital River Laboratories Animal Technology Co. Ltd. (Beijing, China; Permit Number: SCXK (JING) 2012–0001). The animals were housed in standard cages under controlled laboratory conditions (temperature of 22 ± 2 °C, 12-hr light/12-hr dark cycle) with free access to food and water.

### Anesthesia

The anesthesia procedure was performed as previously described [16, 19]. Briefly, the anesthetic chamber was pre-flushed with 30% oxygen in air, with (the isoflurane inhalation group) or without (the control group) 1.33% isoflurane. The concentration of isoflurane, oxygen, and carbon dioxide in the chamber were continuously monitored by gas analyzer (Datex-Ohmeda). When the gas concentration was stable, the mice were placed in the chamber for 1 hour. After anesthesia, mice were returned to their home cage.

### Fear-conditioning tests

All mice were allowed to adapt to the fear-conditioning chamber (Med Associates Inc.) for 7 days (10 mins each day) before the behavioral tests. At 24 hours after isoflurane anesthesia, the mice were allowed to explore the fear-conditioning chamber for 3 minutes, followed by six rounds of fear-conditioning training. One round of training consisted of a 2 Hz pulsating tone (80 dB, 3600 Hz) presented for 30 seconds, followed immediately by a mild foot shock (0.7 mA for 0.5 seconds). Contextual tests were conducted 30 minutes after training, and tonecued tests were conducted 90 minutes after training. For the contextual test, the mice were placed in the same chamber without tone or shock for 3 minutes and their freezing behavior was scored. For the tone-cued test, the mice were placed in an altered chamber with a tone stimulus and their freezing behavior was scored for 6 minutes. The freezing behavior is monitored and analyzed by the observer software (TSE system), and the freezing score is calculated as the ratio of freezing time to total monitoring time.

### Western blot

After the tone-cued test was conducted, the mice were euthanized and their hippocampus tissue was isolated. Western blotting was performed as previously described [20]. The primary antibodies used were α5GABA_A_ (ab10098; Abcam) and GAPDH (5174; Cell Signaling Technology). Western blots were quantified densitometrically using Image Pro-Plus software (Media Cybernetics), and the intensity values of α5GABA_A_ were calculated relative to GAPDH.

### L-655,708 treatment

**Figure 1:**
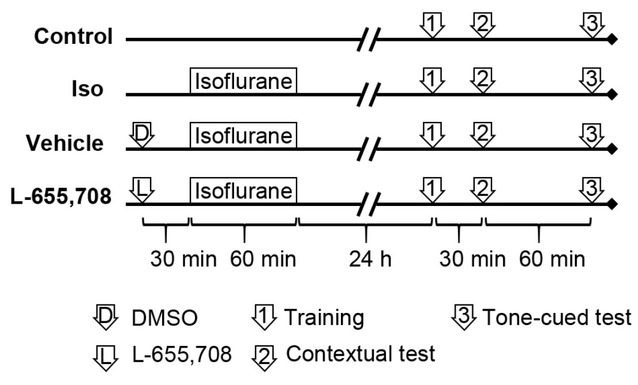
Groups and experimental timelines.

Mice in the L-655,708 group and the vehicle group received intraperitoneal injections of 0.7 mg/kg L-655,708 (130477-52-0; Sigma-Aldrich) or 10% dimethyl sulfoxide (DMSO), respectively. At 30 minutes after the injection of L-655,708 or DMSO, the mice were anesthetized with 1.33% isoflurane for 1 hour, and behavioral tests were conducted 24 hours later, as mentioned earlier. The groups and experimental timelines are depicted in Figure 1.

### Statistical analysis

Quantitative results are expressed as means ± SD. The behavioral data were initially tested for normality using Kolmogorov-Smirnov test with Dallal-Wilkinson-Lillie for corrected P value, and then tested for equal variances using Bartlett’s test. For normally distributed variables, a one-way ANOVA plus a posthoc analysis (Bonferroni test) was used, and for variables not passing a normality or equal variance test, a Kruskal-Wallis test plus a posthoc analysis (Dunn’s multiple comparison test) was used. The quantitative results of the western blots were statistically evaluated using unpaired Student’s *t* tests. All statistical analyses were performed using GraphPad Prism 6.0 software. A P-value <0.05 was considered statistically significant.

## Results

### Old mice exhibited poorer memory than young mice both under physiological conditions and after isoflurane inhalation

**Figure 2:**
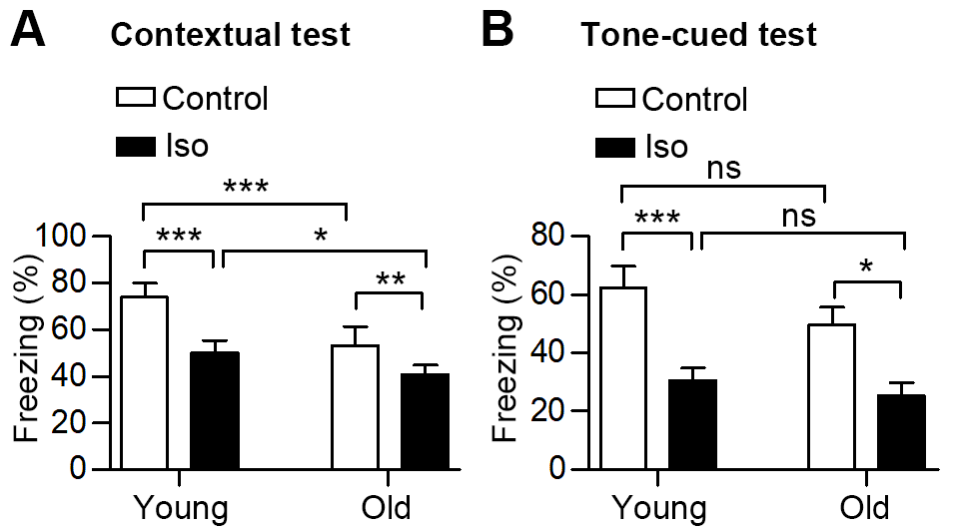
Freezing scores to the context (A) and to the audible cue (B) of the young and old mice under normal conditions and after anesthesia. n = 10 in the groups of young mice; n= 6 in the groups of old mice. All values show means ± SD. *, P<0.05; **, P< 0.01; ***, P<0.001; ns, not significant. P-values were obtained using one-way ANOVA plus Bonferroni posthoc tests in **A** and Kruskal-Wallis test plus Dunn’s multiple comparison test in **B**. Iso, Isoflurane.

To investigate the influence of anesthesia on cognitive function at different ages, we first measured the baseline memory of old and young mice. The FCT is one of the most commonly used behavioral tests to examine cognitive deficits induced by anesthesia, it assesses the hippocampi-dependent (context-related) and hippocampi-independent (tone-related) memory [21, 22]. We found that under physiological conditions, the freezing scores of old mice was notably lower than that of young mice in contextual tests, but not in tone-cued tests (Fig 2, Supplemental Table), indicating

impaired hippocampi-dependent memory in the old mice. We further gave young and old mice an inhalation of 1.33% isoflurane for 1 hour and evaluated their memory 24 hours after anesthesia using FCTs. After isoflurane inhalation, freezing times in both the contextual test and the tone-cued test were significantly decreased in young mice (Fig 2, Supplemental Table). Consistently, the freezing times of old mice were also markedly decreased after anesthesia (Fig 2, Supplemental Table). The difference of the freezing scores after anesthesia between the old and young mice was statistically significant in the contextual tests, while the difference was not remarkable in the tone-cued tests. Taken together, we found that the hippocampi-dependent memory of old mice was worse than that of the young mice under normal conditions and after anesthesia.

### Expression of hippocampal α5GABAA decreased with age

**Figure 3:**
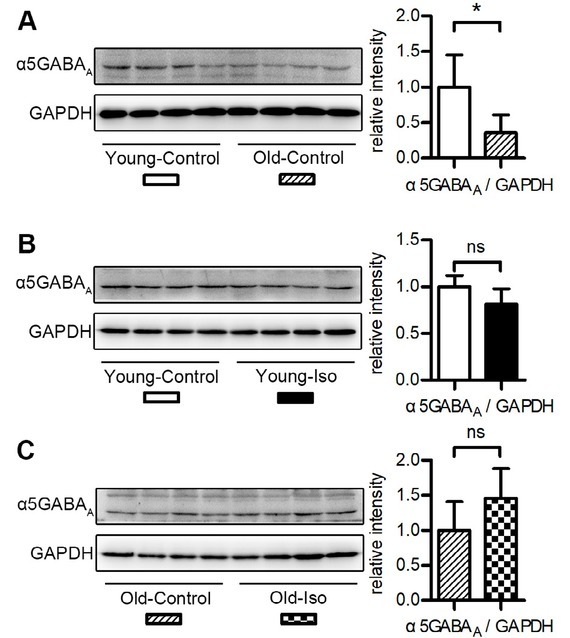
Hippocampal α5GABAA expression in the young and old mice. Quantification of western blots is provided. n = 4 per group. All values show means ± SD. *, P < 0.05; ns, not significant. P-values were obtained using unpaired Student’s t tests. Iso, isoflurane.

The α5GABA_A_ receptors are mainly located in the hippocampus and play a critical role in hippocampi-dependent memory [7, 8]. A previous study suggested that the activity and expression of α5GABA_A_ is increased by anesthetics and contributes to POCD [13]. We therefore wondered whether the expression of α5GABA_A_ increases in old mice, and whether it would be even higher after isoflurane anesthesia. However, western blotting showed that the expression of α5GABA_A_ decreased in the hippocampus of old mice compared with young mice under normal conditions (Fig 3A). Furthermore, a 1-hour exposure to isoflurane did not significantly increase the protein expression of α5GABA_A_ in the hippocampus of both young (Fig 3B) and old mice (Fig 3C). These data suggest that aging causes a decrease in hippocampal α5GABA_A_ expression, but that a 1-hour isoflurane inhalation has no significant effect on the protein expression of α5GABA_A_ in the mouse hippocampus.

### L-655,708 did not improve isoflurane-induced memory deficits in old mice

**Figure 4:**
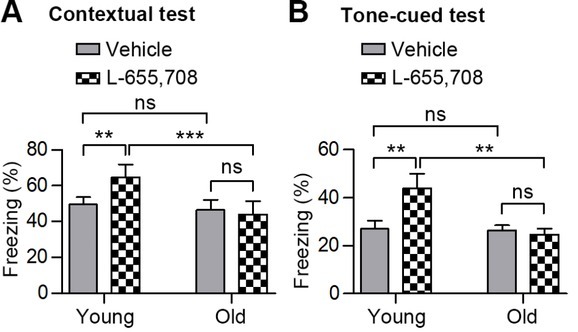
Freezing scores to the context (A) and to the audible cue (B) of the young and old mice pre-injected with L-655,708 or vehicle (DMSO). n = 10 in the groups of young mice; n= 6 in the groups of old mice. All values show means ± SD. **, P< 0.01; ***, P<0.001; ns, not significant. P-values were obtained using Kruskal-Wallis tests plus Dunn’s multiple comparison tests in **A** and **B**.

Studies have shown that the inverse α5GABA_A_ agonist L-655,708 can improve cognitive function by inhibiting the function of α5GABA_A_ [13, 15, 16, 17]. As the expression of α5GABA_A_ decreases with age, we wondered whether the drug might either lose its effect or become more efficient in old mice. We thus gave young and old mice an intraperitoneal injection of 0.7 mg/kg L-655,708 or vehicle (DMSO) 30 minutes before isoflurane anesthesia (1.33% for 1 hour). FCTs were conducted 24 hours later. Consistent with previous studies [13, 16] , the cognitive function of young mice was improved by L-655,708, as evidenced by their increased freezing times when pre-treated with L-655,708 (Fig 4, Supplemental Table). L-655,708 injections however did not significantly reduce the isoflurane-induced memory deficits in old mice (Fig 4, Supplemental Table). Collectively, we observed that, though L-655,708 can prevent isoflurane-induced cognitive decline in young mice, it is not effective in old mice.

## Discussion

In the present study, we evaluated the efficacy of L-655,708, an inverse agonist of α5GABA_A_, in preventing anesthesia-related memory deficits in old mice. We observed that old mice exhibited memory impairment and found that the expression of hippocampal α5GABA_A_ was significantly lower in old mice than in young mice. Furthermore, a 1-hour inhalation of isoflurane caused memory deficits in both young and old mice to a comparable level. L-655,708 alleviated the anesthesia-induced cognitive decline in young mice, but not in old mice. Our study suggests that L-655,708 is not as effective for the prevention of anesthesia-induced cognitive impairment in old mice as it is in young mice.

Age-related cognitive decline is associated with changes of multiple cells, pathways, and molecules [23]. The hippocampus is one of the regions that are more susceptible to aging [24]. We also observed that the hippocampi-dependent memory rather than the hippocampi-independent memory was worse in the old mice than in young mice. Hippocampal CA3 cells in aged rats have abnormally high firing rates compared to the same cells in young rats [25], indicating the hyperactivated state of the hippocampus in aged rats. Correspondingly, it was found that inhibiting the excess activity of CA3 neurons improves cognition in old rats [26]. More importantly, increased hippocampal activation has also been observed in aged humans using functional MRI [27]. These studies implicate that the over-activation, but not the inhibition, of the hippocampus contributes to age-related cognitive impairment. High hippocampal expression of the subunits NR1 and NR2B of N-methyl-D-aspartate receptor, the receptor for excitatory neurotransmitter glutamate, is associated with poorer memory in aged mice [28]. We also find that the protein expression of NR2B in the hippocampus of old mice is higher than that in the young mice under normal conditions (data not shown). Impairment of the GABAergic system, the primary inhibitory system in the central nervous system, is observed in the hippocampus of aged animals, and is closely related to neurocognitive aging [29, 30]. Studies have suggested that the loss of GABAergic interneurons [31, 32], the reduction in GABA receptors [33], the impairment of GABAergic synaptic contacts [34], and the decline in GABA inhibitory postsynaptic currents [35] all contribute to the deterioration of GABAergic function during aging. In our study, old mice exhibited impaired cognition and lower expression of hippocampal α5GABA_A._ Another study also reported a reduction in mRNA levels of α5GABA_A_ in the hippocampus of memory-impaired aged rats [36]. Hence, together with other research, the current findings support the notion that age-related cognitive decline correlates with hippocampal overactivation, which might be caused by the excitatory/inhibitory imbalance.

Studies that use genetically modulated (knockout or point-mutated) mice have suggested that α5GABA_A_ plays a critical role in memory and learning [8, 9, 10]. Plenty of research aimed to use NAMs of α5GABA_A_ receptors to prevent or treat cognitive decline. Inverse agonists of α5GABA_A_, including L-655,708, RO4938581, and α5IA, were reported to enhance cognition without inducing proconvulsant effects in rats [15, 37, 38, 39]; the age of the animals in these studies was however not specifically mentioned. In accordance with the notion that age-related memory decline is accompanied by a reduction in α5GABA_A_, one study showed that TB21007, another NAM of α5GABA_A_, can improve memory performance in young rats, but not in memory-impaired aged rats [40]. More importantly, Compound 6 and Compound 44, two positive allosteric modulators (PAMs) of α5GABA_A_ receptors, were found to be effective in improving cognition in memory-impaired old rats, but not in young rats [40]. As for research in human, α5IA has been found to block alcohol-related learning impairment in healthy young (25-year-old) volunteers [41], while it did not improve performance on a paired-associated learning task (indeed, a dose of 4 mg worsened the performance) in volunteers aged 72 years on average [42]. Age-related memory decline may thus be caused by a decrease, but not by an increase in α5GABA_A_ levels. The different effects of L-655,708 in young and old mice observed in our study also indicate the complex role of α5GABA_A_ in aging. Thus, the use of compounds that regulate α5GABA_A_ receptors to improve cognition in old age needs to be studied further.

The activation of α5GABA_A_ mediates, at least in part, the memory blockade during anesthesia [11, 12, 43]. More importantly, while the synaptic inhibition is transient, tonic inhibition mediated by α5GABA_A_ can persist for weeks after anesthesia [13]. Thus, the persistent increase function of α5GABA_A_ also accounts for persistent memory deficits after general anesthesia [13]. Many studies focus on the use of NAMs of α5GABA_A_ receptors to prevent or to treat cognitive decline caused by anesthesia. Assessed by FCTs and novel object recognition tests, it has been found that pre-injection of L-655,708 can prevent isoflurane-and etomidate-induced memory decline in mice [13, 16, 17]. The age of the animals used in these studies was 8 to 16 weeks [16, 17], or, in one case, not specifically mentioned [13]. We also found that L-655,708 markedly prevents isoflurane-induced cognitive deficits in 3- to 5-month-old mice, while the effect was not obvious in 18- to 20-month-old mice. Several reasons may explain the failure of L-655,708 in preventing anesthesia-induced memory deficits in old mice.

First, as the NAMs of α5GABA_A_ cannot significantly improve cognitive decline in aged animals with low α5GABA_A_ expression [40], the expression levels of α5GABA_A_ may be critical for the efficacy of its NAMs. We found that the expression of α5GABA_A_ was not upregulated by a 1-hour isoflurane inhalation and that the old mice expressed less hippocampal α5GABA_A_. Therefore, we speculate that the relatively low level of α5GABA_A_ even after anesthesia contributed to the ineffectiveness of L-655,708. Overexpression of the α5GABA_A_ receptor in the hippocampus of old mice may help to confirm the role of α5GABA_A_ in the reduced drug effect in old mice. More precise experiments, such as intracerebral injection of α5GABA_A_-expressing lentivirus, are needed for further study.

The second reason may be different responses of old and young brains to anesthesia. There is less new cell proliferation in the hippocampus of aged animals [44, 45], and isoflurane markedly decreased the number of mature neurons in aged, but not young rats [46]. Furthermore, isoflurane induced hippocampal inflammation in aged mice but not young mice [47]. These studies suggest that the alterations caused by anesthetics are amplified in aged brains. Neuroinflammation [48], malfunction of neural stem cells [49], and the activation/ inhibition of ligand-gated ion channels [13, 50, 51] all contribute to neurocognitive impairment after anesthesia. Because of the high susceptibility of the aged to anesthetics, and because various pathways are regulated by anesthetics, we think that there are more changes induced by isoflurane in aged mice; only regulating the α5GABA_A_ pathway may therefore be insufficient to improve cognition.

Third, the activation of other non-α5GABA_A_ receptors by L-655,708 may also have affected its efficacy in our study. The affinity of L-655,708 to the α5 subunit of GABA_A_ receptors is 107, 61, and 54 times that of the α1, α2, and α3 subunits, respectively [52]. It has been shown that a higher dose of inverse agonist may increase the activity of GABA_A_ receptors, because of the agonist-like effects on non-α5GABA_A_ receptors [53]. One study found that L-655,708 at 0.35 mg/kg can fully reverse, while a high dose of L-655,708 (0.7 mg/kg) failed to reverse the memory deficits caused by isoflurane [17], suggesting the excess of L-655,708 might lead to its ineffectiveness. A human study reported that, the maximal tolerated dose of MRK-016, another inverse agonist of α5GABA_A_, is 5 mg in young subjects, while only 0.5 mg in the elderly subjects [54], indicating the effective dose of α5GABA_A_ inverse agonist is much lower in the elderly than in the young one. In the present study, we found that 0.7 mg/kg of L-655,708 can prevent isoflurane-induced memory decline in young mice, but not in the relatively little α5GABA_A_-expressing old mice. Together with previous studies, we speculate that the excess of L-655,708 may activate non-α5GABA_A_ receptors, thus leading to the inefficacy of L-655,708 in the old subjects. Other doses of L-655,708 were not tested in the present study, so it is still unknown whether a lower dose of L-655,708 is effective in old mice.

## Conclusions

We observed that hippocampal α5GABA_A_ expression is lower in old mice than in young mice. Consistent with previous studies, we showed that pretreatment with L-655,708, the inverse agonist of α5GABA_A_, prevented isoflurane-induced cognitive decline in young mice. L-655,708 however did not prevent anesthesia-related memory deficits in old mice. The age-related decrease in α5GABA_A_ in the hippocampus, the vulnerability of the aged brain to anesthetics, and the non-specific GABA activation by L-655,708 might contribute to the difference in the effect of L-655,708 on the prevention of isoflurane-induced memory deficits between young and old mice. It is important to note that the involvement of α5GABA_A_ in anesthesia-induced memory decline in old animals as well as the precise mechanism underlying the failure of L-655,708 to improve cognition in old mice remain to be elucidated. As a number of NAMs of α5GABA_A_ are under development for psychiatric and neurological diseases, special attention should be paid to the different effects in elderly and young patients.

## Abbreviations

POCD: postoperative cognitive dysfunction
GABA: γ-aminobutyric acid subtype A
FCT: fear-conditioning test
NAM: negative allosteric modulator
DMSO: dimethyl sulfoxide
ANOVA: analysis of variance
PAM: positive allosteric modulator.
