# Supplementary material for: L-655,708 Does not Prevent Isoflurane-induced Memory Deficits in Old Mice
Source: Transl Neurosci. 2019 Aug 7;10:180–6. doi: 10.1515/tnsci-2019-0032 (PMC6689210; doi:10.1515/tnsci-2019-0032)
Supplement: Supplementary file 1 [file tnsci-10-180_sm.pdf]

**Supplemental Table. Freezing Scores and statistical analysis of different groups.**

| Groups | | Young-Control | Young-Iso | Old-Control | Old-Iso | Young-Vehicle | Young-L-655,708 | Old-Vehicle | Old-L-655,708 |
|---|---|---|---|---|---|---|---|---|---|
| Contextual tests | n | 10 | 10 | 6 | 6 | 10 | 10 | 6 | 6 |
| | Mean±SD (%) | 73.83±6.246 | 50.07±5.26 | 53.18±8.157 | 40.83±3.991 | 49.55±4.04 | 64.61±7.256 | 46.47±5.717 | 44.06±7.335 |
| | 95% CI (%) | 69.36~78.3 | 46.3~53.83 | 44.62~61.73 | 36.64~45.02 | 46.66~52.44 | 59.42~69.8 | 40.47~52.47 | 36.36~51.75 |
| | Normality[1] | Yes | Yes | Yes | Yes | Yes | Yes | Yes | No |
| | Equal variances[2] | Yes | | | | -- | | | |
| Tone-cued tests | n | 10 | 10 | 6 | 6 | 10 | 10 | 6 | 6 |
| | Mean±SD (%) | 62.26±7.413 | 30.52±4.335 | 49.75±5.946 | 25.3±4.427 | 27.07±3.351 | 43.92±6.048 | 26.32±2.186 | 24.63±2.459 |
| | 95% CI (%) | 56.95~67.56 | 27.42~33.62 | 43.51~55.98 | 20.65~29.94 | 24.68~29.47 | 39.59~48.24 | 24.03~28.61 | 22.05~27.21 |
| | Normality[1] | Yes | No | Yes | Yes | Yes | No | No | Yes |
| | Equal variances[2] | -- | | | | -- | | | |

[1]The normality was tested using Kolmogorov-Smirnov test with Dallal-Wilkinson-Lillie for corrected P value.

[2]Equal variances were tested using Bartlett's test.
